# Rest Intervals during Virtual Reality Gaming Augments Standing Postural Sway Disturbance

**DOI:** 10.3390/s21206817

**Published:** 2021-10-14

**Authors:** Ross Allan Clark, Ancret Szpak, Stefan Carlo Michalski, Tobias Loetscher

**Affiliations:** 1 School of Health and Behavioural Sciences, University of the Sunshine Coast, Sippy Downs, QLD 4556, Australia; 2 Cognitive Aging and Impairment Neurosciences, Justice and Society, University of South Australia, Adelaide, SA 5001, Australia; ancret.szpak@unisa.edu.au (A.S.); stefan.michalski@mymail.unisa.edu.au (S.C.M.); tobias.loetscher@unisa.edu.au (T.L.)

**Keywords:** virtual reality, head-mounted display, balance, postural sway, cybersickness, gaming

## Abstract

Immersive virtual reality (VR) can cause acute sickness, visual disturbance, and balance impairment. Some manufacturers recommend intermittent breaks to overcome these issues; however, limited evidence examining whether this is beneficial exists. The aim of this study was to examine whether taking breaks during VR gaming reduced its effect on postural sway during standing balance assessments. Twenty-five people participated in this crossover design study, performing 50 min of VR gaming either continuously or with intermittent 10 min exposure/rest intervals. Standing eyes open, two-legged balance assessments were performed immediately pre-, immediately post- and 40 min post-exposure. The primary outcome measure was total path length; secondary measures included independent axis path velocity, amplitude, standard deviation, discrete and continuous wavelet transform-derived variables, and detrended fluctuation analysis. Total path length was significantly (*p* < 0.05) reduced immediately post-VR gaming exposure in the intermittent rest break group both in comparison to within-condition baseline values and between-condition timepoint results. Conversely, it remained consistent across timepoints in the continuous exposure group. These changes consisted of a more clustered movement speed pattern about a lower central frequency, evidenced by signal frequency content. These findings indicate that caution is required before recommending rest breaks during VR exposure until we know more about how balance and falls risk are affected.

## 1. Introduction

Immersive virtual reality (VR), defined as a head-mounted display (HMD) that replaces all visual input from the outside world with an interactive artificial environment, holds great promise as an educational, biofeedback and exergaming tool [1,2,3]. However, many VR users develop motion sickness-like symptoms such as nausea, disorientation, and visual disturbances during and after being immersed in VR [1,3,4,5]. VR-induced sickness symptoms may have deleterious effects on hand–eye coordination, cognition, depth perception and visual acuity post-immersion [6,7,8,9]. It may also negatively impact standing balance [7,10], with one study reporting that thirty minutes of VR immersion results in similar balance impairment to that observed when mildly inebriated [11]. In many cases, the negative aftereffects are most prominent immediately after VR immersion and subside within 10–40 min after exiting VR [12,13].

Cybersickness and negative aftereffects have resulted in VR companies providing health and safety recommendations for their users. For example, Facebook Technologies, LLC recommends taking a break at least every 30 min for their popular Oculus Quest 2 headset [14]. The headset manufacturer HTC Technologies refrains in their safety and regulatory guide from providing specific times for the use of their headsets; they simply state that prolonged, uninterrupted use of their headsets should be avoided and that regular breaks should be taken [15]. Considering the advice from VR companies, it is surprising that there is limited publicly available research demonstrating the effectiveness of taking breaks and interrupting prolonged VR exposures on factors such as mitigating cybersickness and standing balance impairment. Some studies found that interrupting VR exposure with short breaks did not stop the development of cybersickness, but led to an increase in cybersickness symptoms with VR exposure time despite taking breaks in between [16,17,18]. However, these studies did not compare a VR exposure with breaks condition with a continuous, uninterrupted VR exposure, and did not focus on standing balance.

This study’s primary aim was to examine the effect of interrupted VR exposure with multiple breaks compared to a long, uninterrupted VR exposure to immersive virtual reality gaming on measures of standing balance, with the center of pressure path length during quiet standing as the primary outcome. Secondary aims were to examine whether alterations, if present, in balance were associated with specific balance control mechanisms via time, time-scale and fractal measures. We hypothesize that breaking up immersive VR gaming with rest periods will attenuate VR impact on postural sway. We also hypothesized that irrespective of interrupted or continuous VR exposure, balance measures would return to baseline levels after 40 min post-VR.

## 2. Materials and Methods

### 2.1. Study Design and Participants

Twenty-five participants (mean age: 23.13 years (SD = 4.50)) enrolled in and completed this study. Participants were all able to move freely during VR exposure, had normal to corrected-to-normal visual acuity and a stereoacuity of at least 63 arcseconds as assessed with the Butterfly Stereo Acuity test. If participants used prescription glasses, they were encouraged to use them throughout the experiment. All participants gave informed consent before participation and were given an honorarium of $20/hour for their participation. Ethics approval for this study was granted by the University of South Australia’s Human Research Ethics Committee (protocol 201073).

### 2.2. Virtual Reality Setup

A commercially available HTC Vive Pro HMD was used to administer a popular game called Space Pirate Trainer (I-Illusions, Geraardsbergen, Belgium). The VR area participants used had dimensions of 3.3 × 3.8 m. A high-performance laptop (Intel Quad Core i7-7820HK processor at 2.90 GHz, 16 GB RAM, Nvidia Geforce GTX 1080 8 GB graphics card) ensured that participants experienced the game at optimal performance. The game required users to combat waves of flying artificially intelligent targets using various virtual weapons, competing for a high score. Users were encouraged to dodge projectiles by moving in three dimensions.

### 2.3. Measures

#### 2.3.1. Balance Measures

Balance measures at each relevant timepoint were derived from a single, 30 s two-legged eyes open standing balance task performed with heels 22 cm apart and toe out angle of 8° on a Nintendo Wii Balance Board. Data were collected via Bluetooth at the natural sampling frequency (≈40 Hz) via a Windows computer. A recent systematic review shows this to be a valid and reliable method of computerized posturography assessment [19].

Posturography data were analyzed using an offline version of the Seesway data analysis program (http://www.rehabtools.org/seesway.html, accessed on 7 July 2021) [20], which resamples the data using cubic spline interpolation to 100 Hz, then low-pass filters at 6.25 Hz using a Coiflet-5 discrete wavelet transform filter. The variables reported in this study have been described in detail with interactive analysis on the site http://www.rehabtools.org/balance-analysis-variables.html, accessed on 7 July 2021, and also in prior literature [21,22,23]. Briefly, the outcome measures consisted of:

*Path length:* The total distance the center of pressure (COP) trace moves throughout the trial. The trace movements can also be expressed as distances along the mediolateral (ML) and anterior–posterior (AP) axes.

*ML and AP path velocity, amplitude and standard deviation:* The axis-specific path velocity, maximum range during the trial for each axis, and standard deviation of the COP trace about the mean COP position.

ML and AP moderate, low, very low and ultralow frequency path length: This splits the COP trace from each axis into moderate (1.56 to 6.25 Hz), low (0.39 to 1.56 Hz), very low (0.10 to 0.39 Hz) and ultralow (<0.10 Hz) frequency signals using a 9-level Symlet-8 wavelet, with the COP path length in each band reported. This results in easily interpretable data, as higher scores reflect more sway.

*ML and AP mean instantaneous frequency and bandwidth:* An analytical wavelet transformation technique, which incorporates the complex Morlet wavelet and is a form of continuous wavelet transform. The frequency bins and time scaling were interpolated to 1 Hz and 10 ms, respectively, allowing for a refined temporal and frequency resolution [24]. The *mean instantaneous frequency* (MIF) and *mean instantaneous bandwidth* (MIB) of the COP axis for each axis were then derived for each timepoint by determining the frequency bin in which the mean wavelet energy value at that timepoint was located (MIF), and the standard deviation of frequencies around that mean value (MIB). This resulted in a MIF and MIB value for every 10 ms epoch in the signals, which was then averaged and reported as the outcome measure. For interpretation, higher MIF reflects a more rapid average speed of postural sway movement; higher MIB represents a greater variety of speeds inherent in the postural sway pattern.

*ML and AP detrended fluctuation analysis (DFA):* A fractal measure assessing complexity via long range correlations in the trace. Practical interpretation of these results is somewhat unclear. However, when DFA < 0.5 or 1 < DFA < 1.5, the signal is anti-persistent (smaller DFA = more anti-persistent). When 0.5 < DFA < 1 or 1.5 < DFA < 2, the signal is persistent (larger DFA = more persistent). Collins, De Luca [25] suggest that greater persistence of the COP data indicates a decline in postural stability, while Amoud, Abadi [26] state that greater anti-persistence reflects a more tightly controlled postural system. DFA alpha 1 possesses a box size between 32 and 100 samples, with DFA alpha 2 including larger box sizes between 100 and 300 samples.

#### 2.3.2. Cybersickness

Self-reports of cybersickness were measured with the Simulator Sickness Questionnaire (SSQ) [27], the most commonly used cybersickness measure in VR research [28].

### 2.4. Procedure

**Figure 1:**
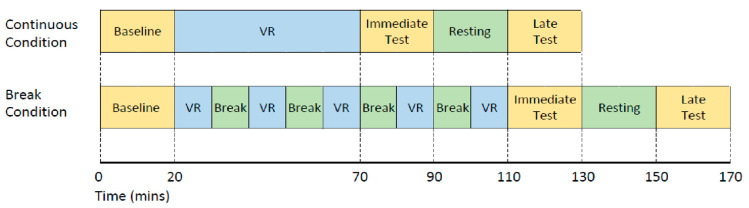
Study design for both the continuous and the break condition completed on different days. VR denotes time exposed to the VR game.

This study utilized a crossover design, with all participants undertaking two VR exposure conditions on two separate days (Figure 1). Once consented, eligible participants were allocated to either start with the continuous or break condition (counterbalanced order). Each condition consisted of a total of 50 min VR. For the continuous VR exposure condition, participants engaged with VR for 50 continuous minutes. In the break condition, participants completed five 10 min VR exposures with four 10 min breaks in between. During these breaks, participants performed static balance assessments which were not assessed. There was a minimum of 24 h between conditions to reduce the influence of residual aftereffects from the previous exposure. There were three relevant measurement timepoints for the purpose of this study:

(1) Baseline: before VR exposure;
(2) Immediate: immediately after completing a total of 50 min VR;
(3) Late: 40 min after exiting VR exposure. The 40 min post-VR included a 20 min resting time between the immediate and late test.

At each of the three timepoints in either condition, balance assessments and SSQ data were collected along with other tests including a range of cognitive measures which are not described here.

### 2.5. Statistical Analysis

#### 2.5.1. Primary Aim

For COP path length (primary aim), a repeated-measures ANOVA with the factors Condition (Continuous and Breaks) and Timepoint (Baseline, Immediate, Late) was conducted. Greenhouse–Geisser sphericity corrections were applied in cases where the sphericity assumption was violated. Holm–Bonferroni-adjusted post hoc tests were conducted for (1) comparisons between the conditions at the three timepoints, and (2) comparison of the different timepoints within each condition.

#### 2.5.2. Secondary Aims

For the other balance variables (secondary aims), targeted paired-samples *t*-tests and Cohen’s d effect size statistical comparisons were performed for (1) comparisons between the conditions at the three timepoints, and (2) for comparison within each condition for a possible difference between Baseline and Immediate, as well as Baseline and Late timepoints. To reduce the risk of Type 1 error given the large number of analyses performed, and due to the exploratory nature of the secondary aims, to be deemed important, these variables must be both *p* < 0.05 and the effect size > 0.83. This effect size value was chosen based on the available sample size, allowing statistically significant differences across timepoints to be confidently identified at alpha = 0.05 and power = 0.80.

Self-reports of cybersickness, as measured via the SSQ, were also submitted to a repeated-measures ANOVA with the factors Condition (Continuous and Breaks) and Timepoint (Baseline, Immediate, Late) conducted. Holm–Bonferroni-adjusted post hoc tests were conducted in the case of significant interactions.

## 3. Results

### 3.1. Primary Aim 

**Figure 2:**
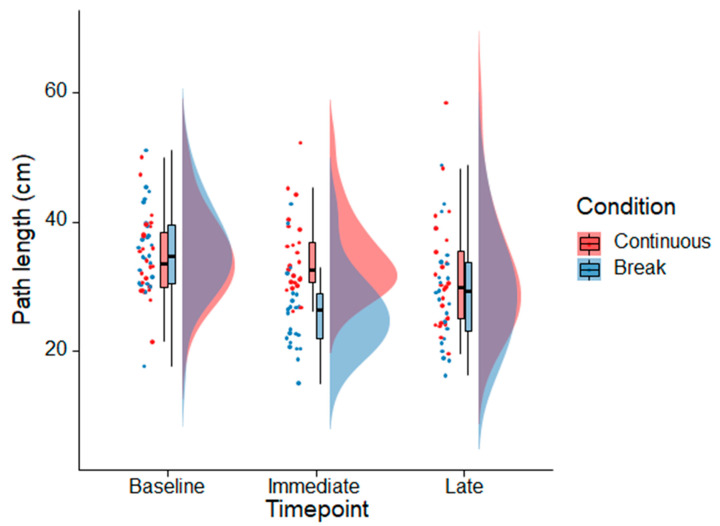
Raincloud plots [29] showing the distributions of the path length data for the continuous and break VR conditions before VR exposure (baseline), immediately after a total of 50 min of VR, and 40 min after the last VR exposure (late timepoint). The dots show individual data observations. The boxplots indicate the conditions’ median, upper and lower quartiles, as well as the distance of 1.5× the interquartile range from the upper and lower quartiles, respectively (whiskers). The half violin plots display a probability density function of the data see [29] for more information.

For the primary outcome measure path length, there was a main effect of *Condition* (F(1,22) = 10.23, *p* = 0.004, partial η^2^ = 0.317), with lower path length in the break than the continuous condition. There was also a main effect of *Timepoint* (F(1.5,33.7) = 16.917, *p* < 0.001, partial η^2^ = 0.435) and a significant interaction between *Condition* and *Timepoint* (F(2,44) = 11.377, *p* < 0.001, partial η^2^ = 0.341). Holm–Bonferroni-adjusted post hoc tests revealed a lower path length for the break than continuous VR condition immediately after a total of 50 min VR (*p* < 0.001, Cohen’s d = 1.049). There were no significant differences between the conditions at baseline (*p* = 0.732, Cohen’s d = −0.118) and at the late timepoint (*p* = 0.312, Cohen’s d = 0.346). See Figure 2.

In the break VR condition, path length decreased from baseline to immediately after VR (*p* < 0.001, Cohen’s d = 1.255). Compared to baseline, path length was still decreased at the late timepoint (*p* < 0.001, Cohen’s d = 0.825). In contrast, in the continuous condition there were no statistically significant differences from baseline to the immediate timepoint (*p* = 0.747, Cohen’s d = 0.067) and from baseline to the late timepoint (*p* = 0.104, Cohen’s d = 0.485).

### 3.2. Secondary Aims

**Figure 3:**
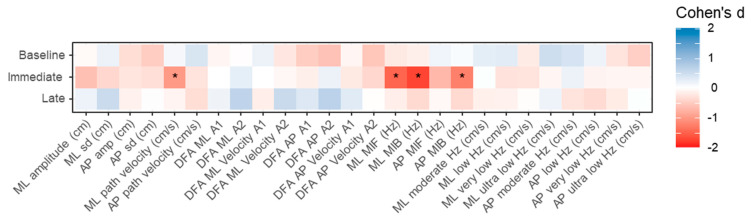
Heatmaps indicating the Cohen’s d for differences between the continuous and break VR conditions for the different balance measures at the three measurement timepoints. * indicates an effect size smaller than −0.83 or larger than 0.83. ML: medial–lateral; AP: anterior–posterior; DFA: detrended fluctuation analysis; MIF: mean instantaneous frequency; MIB: mean instantaneous bandwidth.

The effect sizes (Cohen’s d) for the comparisons of the different balance measures between the continuous and break conditions at the three different timepoints are provided in Figure 3. Differences with effect sizes larger than >0.83 between the two conditions were only found for 4 variables immediately after VR exposure. Namely, ML path velocity (Cohen’s d = 1.01, *p* < 0.001), ML MIF (Cohen’s d = 1.50, *p* < 0.001), ML MIB (Cohen’s d = 1.75, *p* < 0.001) and AP MIB (Cohen’s d = 1.24, *p* < 0.001). There were no differences between the conditions with effect sizes larger than 0.83 at baseline and late assessments.

**Figure 4:**
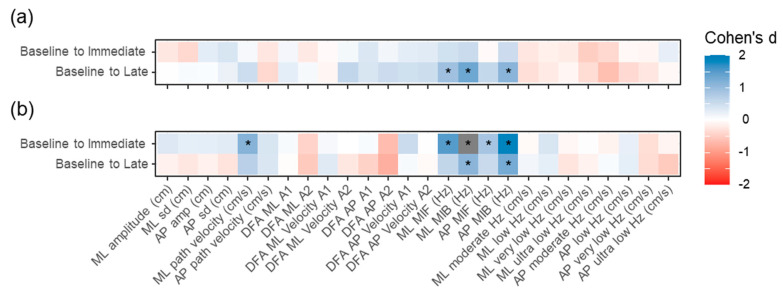
Heatmaps indicating the Cohen’s d for differences between baseline and later measures for (**a**) the continuous VR and (**b**) the break condition. * indicates an effect size smaller than −0.83 or larger than 0.83. ML: medial–lateral; AP: anterior–posterior; DFA: detrended fluctuation analysis; MIF: mean instantaneous frequency; MIB: mean instantaneous bandwidth.

Figure 4 depicts the comparison within each condition for possible difference between baseline and later timepoints. No statistically significant differences with Cohen’s d > 0.83 were observed in the continuous exposure condition between baseline and immediate post-VR measurements (Figure 4b). However, at the late timepoint, statistically significant differences which exceeded the Cohen’s d > 0.83 threshold were observed for ML MIF (Cohen’s d = 0.93, *p* = 0.001), ML MIB (Cohen’s d = 1.33, *p* < 0.001) and AP MIB (Cohen’s d = 1.11, *p* < 0.001).

For the break condition (Figure 4b), five variables had statistically significant (*p* < 0.05) effect size values > 0.83 between the Baseline and Immediate timepoint assessment; ML path velocity (Cohen’s d = 1.15, *p* < 0.001), ML MIF (Cohen’s d = 1.49, *p* < 0.001), ML MIB (Cohen’s d = 2.15, *p* < 0.001), AP MIF (Cohen’s d = 0.85, *p* < 0.004), AP MIB (Cohen’s d = 1.88, *p* < 0.001). Four of these five variables were also statistically significantly different from the baseline measures at the late timepoint. However, the magnitude was attenuated for all five, with only ML MIB (Cohen’s d = 1.10, *p* < 0.001) and AP MIB (Cohen’s d = 1.27, *p* < 0.001) having effect sizes larger than 0.83.

For self-reports of cybersickness (i.e., SSQ), there was a main effect of *Timepoint* (F(2,46) = 13.93, *p* < 0.001, partial η^2^ = 0.377), with SSQ scores being higher after the immediate timepoint (M = 20.03, SE = 3.04) compared to the baseline (M = 10.75, SE = 3.04) (*p* < 0.001) and the late timepoint (M = 10.28, SE = 3.04) (*p* < 0.001). Both the continuous (M = 10.44, SE = 3.29) and break (M = 11.06, SE = 3.29) conditions had similar pre-exposure scores, which were not significantly different immediately after VR (continuous (M = 21.82, SE = 3.29) and break (M = 18.23, SE = 3.29) conditions) and at the late timepoint SSQ scores returned to baseline levels (continuous (M = 10.13, SE = 3.29) and break (M = 10.44, SE = 3.29) conditions). There was no main effect of *Condition* (F(1,23) = 0.236, *p* = 0.632, partial η^2^ = 0.010) and no significant interaction between *Condition* and *Timepoint* (F(2,46) = 1.21, *p* = 0.308, partial η^2^ = 0.050). The severity of SSQ symptoms according to [27] can be categorized as no/minimal symptoms (0–10), significant symptoms (11–20) and symptoms of serious concern (>20) also see [9].

## 4. Discussion

To our knowledge, this is the first study that directly compared the impact of implementing intermittent rest periods on postural sway patterns to uninterrupted, continuous VR gaming. Our primary finding was that short-duration, repetitive exposure broken up by rest periods may augment changes in standing balance immediately after immersion relative to continuous exposure. These adaptations may persist even after an extended rest interval, as shown in the Baseline versus Late timepoint analysis. Our secondary findings were that these changes consist of a more clustered movement speed pattern (evidenced by reduced MIB) about a lower central frequency (evidenced by reduced MIF). Whilst the effect size of these axis-specific MIF and MIB changes were very high in the break condition group, an inspection of the discrete-wavelet bandwidth variables indicates that this resulted in a subtle shift with less movement at higher frequencies and more occurring at lower frequencies. However, within each individual bandwidth, these were typically not significant differences, and in instances where they were, the effect size was modest. As such, it does not appear to be a single bandwidth (i.e., movement speed) dominating the adaption, but a general widespread change in the movement pattern.

Our findings were unexpected and did not support our hypothesis. In the break condition, participants had to switch between the real and virtual world every 10 min. HMDs induce accommodation–vergence [8,9] and visual–vestibular conflicts [3]. It will be an important direction for further study whether these repeated adaptations to altered motor–sensory environments underlie the observed changes in postural sway.

Moreover, we do not know if the observed changes are beneficial or an impairment. In clinical populations, a reduction in COP path length as observed in our study is often considered a beneficial effect; for example, reduced total COP path length is associated with increased walking ability [30] and chair rise ability [31] in people living with stroke. Additionally, less variation in postural sway has been associated with greater levels of cybersickness in a study with healthy participants [32]. Conversely, postural instability has been linked to cybersickness susceptibility [33,34,35]. The self-reports of cybersickness obtained in this study do not indicate whether the path length changes in the break condition are indicative of a negative effect. The observed increase in cybersickness after 50 min VR was not modulated by the VR exposure condition. As such, whilst we are confident that alteration does occur in response to VR exposure and that interrupting VR exposure appears to augment the effect, we cannot determine its consequences. Individual characteristics contributing to cybersickness, such as sex, age and health status, are yet to be understood. This study cannot contribute to the understanding of individual characteristics. For example, there were only nine females recruited in this study, which is too small to examine sex differences in cybersickness and postural adaptations. Further research in cybersickness and postural alterations is therefore needed, particularly studies that attempt to perturb balance post-VR exposure.

Our study had limitations. Firstly, we possessed a relatively small sample size given the number of analyses performed. To counter this, we imposed a relatively strict effect size threshold based on a sample size calculation for our exploratory secondary outcome measures. Additionally, this did not allow us to perform further analyses comparing all timepoints. However, despite these accommodations, we cannot discount that some of the findings were due to random chance. Secondly, in the break condition, participants performed balance assessments during each of the breaks between VR immersion, and as such, performed more assessments than the continuous group between the timepoints used in our statistical analysis. This may have resulted in a learning effect. However, prior research has shown that COP variables related to velocity, amplitude and entropy collected using a WBB are stable and show no learning effect in healthy or visually-impaired adults when performed at 15 min intervals [36]. Thirdly, our cohort consisted of young, healthy people. Results may vary in other populations. Fourthly, our VR immersion experience was a fast-paced game requiring high levels of upper body movement. Whilst we believe that this represents a typical gaming scenario, whether a similar finding for postural sway alteration is present in more slow-placed educational VR immersion is unknown. To support this, a recent study [37] indicates that in healthy young and older adults and those with Parkinson’s disease, performing a simple walking task while immersed in VR has no impact on COP sway area measured post-immersion. Conversely, another study observed that the speed and direction of movement during VR immersion did not impact postural sway patterns [38].

## 5. Conclusions

Caution should be implemented if using immersive VR in high-fall-risk populations until we know more about how balance is affected. Breaking up immersion may increase alterations in sway patterns. Thus, it appears that it is not a mechanism for attenuating possible adverse effects of VR immersion on postural coordination. However, further research is required.

## Data Availability

The data presented in this study are available on request from the corresponding author.

## References

[1] Jerald J. (2016). The VR Book: Human-Centered Design for Virtual Reality.

[2] Bailenson J. (2018). Experience on Demand: What Virtual Reality Is, How It Works, and What It Can Do.

[3] Hale K., Stanney K. (2014). Handbook of Virtual Environments.

[4] Saredakis D., Szpak A., Birckhead B., Keage H.A., Rizzo A., Loetscher T. (2020). Factors Associated With Virtual Reality Sickness in Head-Mounted Displays: A Systematic Review and Meta-Analysis. Front. Hum. Neurosci..

[5] Chang E., Kim H.T., Yoo B. (2020). Virtual Reality Sickness: A Review of Causes and Measurements. Int. J. Hum.–Comput. Interact..

[6] Muth E.R. (2009). The challenge of uncoupled motion: Duration of cognitive and physiological aftereffects. Hum. Factors.

[7] Champney R.K., Stanney K.M., Hash P.A., Malone L.C., Kennedy R.S., Compton D.E. (2007). Recovery from virtual environment exposure: Expected time course of symptoms and potential readaptation strategies. Hum. Factors.

[8] Hoffman D.M., Girshick A.R., Akeley K., Banks M.S. (2008). Vergence–accommodation conflicts hinder visual performance and cause visual fatigue. J. Vis..

[9] Szpak A., Michalski S.C., Saredakis D., Chen C.S., Loetscher T. (2019). Beyond Feeling Sick: The Visual and Cognitive Aftereffects of Virtual Reality. IEEE Access.

[10] Hakkinen J., Vuori T., Paakka M. Postural stability and sickness symptoms after HMD use. Proceedings of the IEEE International Conference on Systems, Man and Cybernetics.

[11] Kennedy R.S., Lilienthal M.G. Implications of balance disturbances following exposure to virtual reality systems. Proceedings of the IEEE Virtual Reality Annual International Symposium (VRAIS).

[12] Dużmańska N., Strojny P., Strojny A. (2018). Can simulator sickness be avoided? A review on temporal aspects of simulator sickness. Front. Psychol..

[13] Szpak A., Michalski S.C., Loetscher T. (2020). Exergaming With Beat Saber: An Investigation of Virtual Reality Aftereffects. J. Med. Internet Res..

[14] Facebook Technologies LLC Oculus Quest 2-Health and Safety Warnings. https://www.oculus.com/legal/health-and-safety-warnings/.

[15] HTC Technologies Safety and Regulatory Guide. https://dl4.htc.com/vive/safty_guide/91H02887-08M%20Rev.A.PDF?_ga=1.174055779.1348312037.1468207042.

[16] Deb S., Carruth D.W., Sween R., Strawderman L., Garrison T.M. (2017). Efficacy of virtual reality in pedestrian safety research. Appl. Ergon..

[17] Lampton D.R., Rodriguez M.E., Cotton J.E. (2000). Simulator Sickness Symptoms during Team Training in Immersive Virtual Environments. Proc. Hum. Factors Ergon. Soc. Annu. Meet..

[18] Moss J.D., Austin J., Salley J., Coats J., Williams K., Muth E.R. (2011). The effects of display delay on simulator sickness. Displays.

[19] Clark R.A., Mentiplay B.F., Pua Y.H., Bower K.J. (2018). Reliability and validity of the Wii Balance Board for assessment of standing balance: A systematic review. Gait Posture.

[20] Clark R.A., Pua Y.H. (2018). SeeSway–A free web-based system for analysing and exploring standing balance data. Comput. Methods Programs Biomed..

[21] Clark R.A., Howells B., Feller J., Whitehead T., Webster K.E. (2014). Clinic-based assessment of weight-bearing asymmetry during squatting in people with anterior cruciate ligament reconstruction using nintendo wii balance boards. Arch. Phys. Med. Rehabil..

[22] Clark R.A., Seah F.T., Chong H.C., Poon C.L., Tan J.M., Mentiplay B.F., Pua Y.H. (2017). Standing balance post total knee arthroplasty: Sensitivity to change analysis from four to twelve weeks in 466 patients. Osteoarthr. Cartil..

[23] Liang Z., Clark R., Bryant A., Quek J., Pua Y.H. (2014). Neck musculature fatigue affects specific frequency bands of postural dynamics during quiet standing. Gait Posture.

[24] Bryant A.L., Pua Y.H., Clark R.A. (2009). Morphology of knee extension torque-time curves following anterior cruciate ligament injury and reconstruction. J. Bone Jt. Surg.-Ser. A.

[25] Collins J.J., De Luca C.J., Burrows A., Lipsitz L.A. (1995). Age-related changes in open-loop and closed-loop postural control mechanisms. Exp. Brain Res..

[26] Amoud H., Abadi M., Hewson D.J., Michel-Pellegrino V., Doussot M., Duchêne J. (2007). Fractal time series analysis of postural stability in elderly and control subjects. J. NeuroEng. Rehabil..

[27] Kennedy R.S., Lane N.E., Berbaum K.S., Lilienthal M.G. (1993). Simulator Sickness Questionnaire: An Enhanced Method for Quantifying Simulator Sickness. Int. J. Aviat. Psychol..

[28] Rebenitsch L., Owen C. (2016). Review on cybersickness in applications and visual displays. Virtual Real..

[29] Allen M., Poggiali D., Whitaker K., Marshall T.R., Kievit R.A. (2021). Raincloud plots: A multi-platform tool for robust data visualization. Wellcome Open Res..

[30] Mentiplay B.F., Williams G., Tan D., Adair B., Pua Y.H., Bok C.W., Bower K.J., Cole M.H., Ng Y.S., Lim L.S. (2019). Gait Velocity and Joint Power Generation after Stroke: Contribution of Strength and Balance. Am. J. Phys. Med. Rehabil..

[31] Mentiplay B.F., Clark R.A., Bower K.J., Williams G., Pua Y.H. (2020). Five times sit-to-stand following stroke: Relationship with strength and balance. Gait Posture.

[32] Dennison M.S., D’Zmura M. (2017). Cybersickness without the wobble: Experimental results speak against postural instability theory. Appl. Ergon..

[33] Riccio G.E., Stoffregen T.A. (1991). An ecological Theory of Motion Sickness and Postural Instability. Ecol. Psychol..

[34] Risi D., Palmisano S. (2019). Effects of postural stability, active control, exposure duration and repeated exposures on HMD induced cybersickness. Displays.

[35] Chardonnet J.-R., Mirzaei M.A., Mérienne F. (2017). Features of the Postural Sway Signal as Indicators to Estimate and Predict Visually Induced Motion Sickness in Virtual Reality. Int. J. Hum.–Comput. Interact..

[36] Jeter P.E., Wang J., Gu J., Barry M.P., Roach C., Corson M., Yang L., Dagnelie G. (2015). Intra-session test-retest reliability of magnitude and structure of center of pressure from the Nintendo Wii Balance Board™ for a visually impaired and normally sighted population. Gait Posture.

[37] Kim A., Darakjian N., Finley J.M. (2017). Walking in fully immersive virtual environments: An evaluation of potential adverse effects in older adults and individuals with Parkinson’s disease. J. NeuroEng. Rehabil..

[38] Widdowson C., Becerra I., Merrill C., Wang R.F., LaValle S. (2019). Assessing Postural Instability and Cybersickness Through Linear and Angular Displacement. Hum. Factors.

